# Highly Sensitive Marker Panel for Guidance in Lung Cancer Rapid Diagnostic Units

**DOI:** 10.1038/srep41151

**Published:** 2017-01-24

**Authors:** Sonia Blanco-Prieto, Loretta De Chiara, Mar Rodríguez-Girondo, Lorena Vázquez-Iglesias, Francisco Javier Rodríguez-Berrocal, Alberto Fernández-Villar, María Isabel Botana-Rial, María Páez de la Cadena

**Affiliations:** 1 Department of Biochemistry, Genetics and Immunology, Faculty of Biology, Universidad de Vigo. 36310 Vigo, Spain; 2 Department of Medical Statistics and Bioinformatics, Leiden University Medical Center. 2300RC Leiden, The Netherlands; 3 SiDOR Research Group & Centro de Investigaciones Biomédicas (CINBIO), Faculty of Economics and Business Administration, Universidad de Vigo. 36310 Vigo, Spain; 4 Servicio de Neumología Hospital Álvaro Cunqueiro EOXI Vigo, Instituto de Investigación Biomédica de Vigo. 36312 Vigo, Spain

## Abstract

While evidence for lung cancer screening implementation in Europe is awaited, Rapid Diagnostic Units have been established in many hospitals to accelerate the early diagnosis of lung cancer. We seek to develop an algorithm to detect lung cancer in a symptomatic population attending such unit, based on a sensitive serum marker panel. Serum concentrations of Epidermal Growth Factor, sCD26, Calprotectin, Matrix Metalloproteinases −1, −7, −9, CEA and CYFRA 21.1 were determined in 140 patients with respiratory symptoms (lung cancer and controls with/without benign pathology). Logistic Lasso regression was performed to derive a lung cancer prediction model, and the resulting algorithm was tested in a validation set. A classification rule based on EGF, sCD26, Calprotectin and CEA was established, able to reasonably discriminate lung cancer with 97% sensitivity and 43% specificity in the training set, and 91.7% sensitivity and 45.4% specificity in the validation set. Overall, the panel identified with high sensitivity stage I non-small cell lung cancer (94.7%) and 100% small-cell lung cancers. Our study provides a sensitive 4-marker classification algorithm for lung cancer detection to aid in the management of suspicious lung cancer patients in the context of Rapid Diagnostic Units.

Lung cancer (LC) is the most common cause of cancer-related death worldwide, accounting for 13% of new cancer diagnoses and 19% of total deaths^1^. Despite recent advances in treatment, this neoplasia carries an extremely poor prognosis, with an overall 5-year survival rate of 13% 2, consequence of the difficulty of detection at an early stage^3^. Therefore, early detection when surgery may be curative is the best way to reduce LC mortality^2^,^4^.

Opposed to the scenario in U.S., where screening using low-dose computed tomography (CT) among high-risk individuals has been recommended^5^, in Europe there are no LC screening recommendations so far^6^. Among the main reasons are the high rate of false positive results^7^,^8^,^9^, and the awaited upcoming results of the on-going randomized control trials (reviewed in Ruchalski *et al*.^9^).

In Spain, the reduction of the time before diagnosis and staging is a priority^8^,^10^,^11^,^12^, since both radiological imaging (CT, PET) and invasive procedures for histological confirmation (bronchoscopy, thoracic needle aspiration or thoracentesis) are required in the diagnostic work-up of a LC patient^13^,^14^. Rapid or Quick Diagnostic Units (RDU/QDU) have been established within the public health system, with the main objective of accelerating the early diagnosis of potentially severe diseases such as cancer, avoiding hospitalisations for purely diagnostic purposes, minimizing hospital-related morbidity, reducing costs, and improving patient satisfaction^15^,^16^. In Spain approximately 40–50% of the patients attending LC-RDUs display non-cancerous lung pathologies^11^,^12^. Consequently, clinical decision-making in the setting of LC-RDU would benefit from non-invasive markers that could help predict LC risk in symptomatic individuals, discerning cancerous patients who should be submitted to confirmatory diagnostic procedures from those without cancer in whom a conservative approach could be applied avoiding initially such procedures. Recent reviews have indicated that blood-based markers would be an ideal tool to detect early-stage LC and complement CT imaging^17^,^18^.

We previously described a three-marker panel that included EGF (Epidermal Growth Factor), soluble CD26 (sCD26) and Calprotectin (CAL), showing a considerable discriminatory capacity to detect patients at high risk for LC (83% sensitivity and 87% specificity)^19^. In this new study, together with EGF, sCD26, and CAL, other 5 serum markers were evaluated: MMP-1, −7, −9 (Matrix Metalloprotease −1, −7, −9), CEA (Carcinoembryonic antigen) and CYFRA 21.1, with the aim of improving our previous diagnostic algorithm. These molecules cover a spectrum of biological functions implicated in cancer development and progression, as summarized on Table 1. Since this novel panel is intended to be used in a LC-RDU managed by consultants receiving referrals from primary care doctors, an elevated sensitivity to detect LC among symptomatic patients is imperative.

## Results

### Marker Levels in Lung Cancer and Controls

Serum levels of the 8 markers analyzed are shown in Table 2, including the median and range for the control group (healthy and benign) and LC. After correction for multiple testing, serum concentrations of EGF, CAL, MMP-1, MMP-7, MMP-9, CEA and CYFRA 21.1 were significantly elevated in LC compared to controls (Mann-Whitney U test, *P* = 0.001 for EGF, CAL, MMP-9, CEA and CYFRA 21.1; *P* = 0.047 for MMP-1 and *P* = 0.013 for MMP-7), while sCD26 levels were notably lower in malignancy relative to controls (Mann-Whitney U test, *P* = 0.001).

All marker levels were found significantly different between healthy controls and cancer subjects (Mann-Whitney U test, *P* = 0.002 for EGF, sCD26, CAL, MMP-9, CEA and CYFRA 21.1; *P* = 0.018 for MMP-1 and MMP-7). However, when comparing patients with benign pathologies and cancer, differences in MMP-1 and MMP-7 resulted not significant (Mann-Whitney U test, *P* = 0.448 for MMP-1 and *P* = 0.090 for MMP-7). In multivariate linear regression models adjusted for gender, age and smoking status, significant association was again observed for the occurrence of LC and the markers, except for MMP-1 and MMP-7, when considering both the healthy group and all controls. However, only CAL, MMP-9 and CEA maintained significant association with LC regarding benign pathologies.

Furthermore, correlation between the eight markers analysed was also explored using an annotated heatmap (Supplementary Figure S1). Correlations rank from a minimum of 0.038 between EGF and CYFRA 21.1, and a maximum of 0.489 for CAL and MMP-9. Moderate correlations were also observed for several markers, as with EGF with CAL and MMP-9, and the negative correlation of sCD26 with CAL.

The performance of the candidate markers was evaluated by means of ROC curves (Table 2). CAL showed the best potential to discriminate LC from controls (AUC 0.759), followed by CEA (AUC 0.744), CYFRA 21.1 (AUC 0.734) and MMP-9 (AUC 0.729). EGF and sCD26 exhibited AUCs in the range of 0.7, while MMP-1 and MMP-7 demonstrated poor discriminatory capacity (AUC 0.597 and 0.627, respectively).

### Marker Levels by Cancer Histology and Stage

As displayed in Table 3, LC cases were evaluated based on histology. Statistically significant differences after correction for multiple testing were found between both non-small cell lung cancer (NSCLC) and small cell lung cancer (SCLC) in relation to controls for sCD26, CEA and CYFRA 21.1 (Mann Whitney U test, NSCLC vs controls: *P* = 0.002 for sCD26, CEA and CYFRA 21.1; SCLC vs controls: *P* = 0.040 for sCD26, *P* = 0.002 for CEA and *P* = 0.012 for CYFRA 21.1). Levels of EGF, CAL, MMP-7 and MMP-9 resulted different when comparing NSCLC and controls (Mann-Whitney U test, *P* = 0.002 for EGF, CAL and MMP-9, *P* = 0.048 for MMP-7), but not for SCLC (Mann-Whitney U test, *P* = 0.798 for EGF and MMP-9, *P* = 0.112 for CAL and *P* = 0.056 for MMP-7).

The potential of the markers to detect early stage LC was analysed according to tumour stage (Table 3). EGF, CAL and MMP-9 were the only molecules significantly altered after multiple testing correction in NSCLC stage I + II (Mann-Whitney U test, *P* = 0.002 for EGF and MMP-9, *P* = 0.017 for CAL), suggesting their usefulness for diagnosis at earliest stages. For late stage NSCLC, all markers except for MMP-1 displayed significant differences (Mann-Whitney U test, *P* = 0.002 for EGF, sCD26, CAL, MMP-9, CEA and CYFRA 21.1, *P* = 0.023 for MMP-7). Regarding SCLC a dramatic reduction of EGF, CAL and MMP-9 levels in limited SCLC prevents the distinction from non-cancer patients. After adjusting for the common risk factors gender, age and smoking, the same associations were maintained, with the exception of SCLC association with sCD26, and the lack of significance with either LC histology or NSCLC stages and MMP-7.

### Association between Clinical Parameters and Marker Levels

The association of marker concentrations with clinical variables gender, age and smoking status is presented in Supplementary Table S1. sCD26 levels were significantly higher in women relative to men (Mann-Whitney U test, *P* = 0.018), with no other marker influenced by gender. Older age was associated significantly with higher levels of MMP-7 and CYFRA 21.1 (Mann-Whitney U test, *P* < 0.001 and *P* = 0.004, respectively), whereas sCD26 levels diminished with age. Patients with smoking habits had significantly increased serum concentrations of EGF and MMP-7 in relation to never smokers (Mann-Whitney U test, *P* = 0.028 for EGF and *P* = 0.026 for MMP-7).

### Multimarker Panel and Classification Algorithm for Lung Cancer

Lasso regression was employed to simultaneously derive a multivariate panel of markers and an optimal cut-off for LC, with the criterion of maximizing specificity for a predefined sensitivity of 95%. The resulting classification rule as well as the optimal Lasso penalization parameter are available in Supplementary Material S1 and Supplementary Figure S2, respectively. Additionally, Supplementary Table S2 includes several diagnostic measurements for the proposed model.

Variability in the proportion of males and smoking, and differences in age between LC and controls, as well as influence of these variables on marker levels motivated their inclusion in the model. Application of Lasso procedure on the training set led to the establishment of a 4-marker panel composed of EGF, sCD26, CAL and CEA. A clinical model composed of gender, age and smoking was also established by logistic regression for comparison.

**Figure 1:**
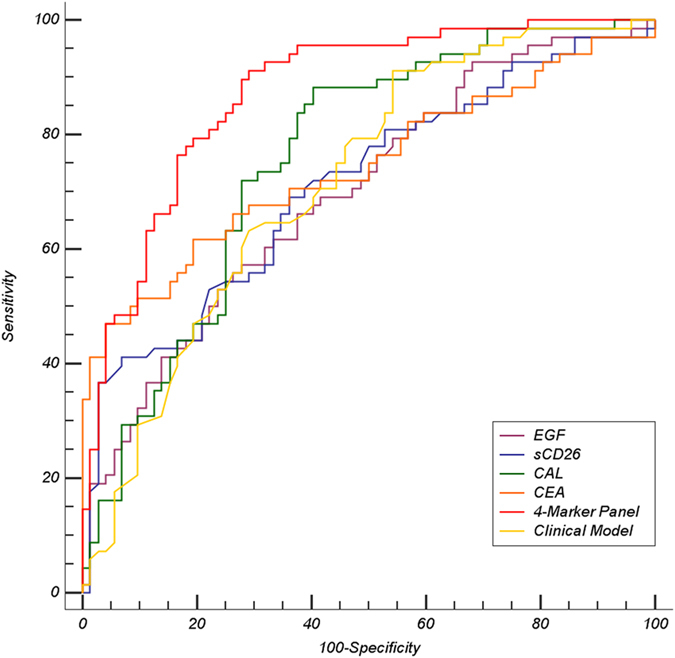
ROC Curve Analysis for Lung Cancer Prediction in the Training Set. ROC curves are shown for each individual marker included in the classification algorithm, together with the clinical model and the 4-marker panel derived from logistic Lasso regression. Training set included 68 lung cancer cases and 72 controls (36 healthy and 36 benign respiratory pathologies).

Performance and ROC curves of this marker panel and single markers for LC diagnosis, besides the clinical model, are presented on Table 4 and Fig. 1, respectively. The 4-marker panel demonstrated a good discriminatory capacity to differentiate LC patients from controls with an AUC of 0.873, showing 97% sensitivity and 43% specificity for LC detection corresponding to a 0.266 cut-off. This combination of markers outperforms the individual markers in terms of specificity. In relation to the clinical model, a lower discriminatory ability was displayed as compared to our proposed multivariate panel (AUC = 0.717 (0.637–0.799), DeLong test *P*-value < 0.0001). At the desired sensitivity of 95%, the decision rule based on such clinical model renders a poor specificity of 26%. Based on our model and assuming a prevalence of LC of 44.4%, corresponding to the RDU of the Pneumology Service of EOXI Vigo, an optimal Negative Predictive Value (NPV) of 94.7% was reached, and a moderate Positive Predictive Value (PPV) of 57.6%.

To further verify the performance of the 4-marker panel for prediction of LC, the resulting classification algorithm developed in the training set was tested in an independent validation set. Descriptive statistics for each marker of the panel are given in Supplementary Table S3 according to histology and stage. In the validation set the marker panel showed an AUC of 0.837, with a sensitivity of 91.7% and a moderately higher specificity of 45.4%, based on the 0.266 cut-off established (Table 4). Regarding the clinical model, the inferior discriminatory capacity was again evidenced by the AUC of 0.659 (0.488–0.816) (DeLong test *P*-value = 0.0003).

### Classification Accuracy of the 4-Marker Panel for Lung Cancer and Control Subgroups

To deeply assess the performance of our classification rule we examined its ability to correctly classify specific subgroups of LC patients and controls (Table 5). Training and validation populations were combined, and sensitivity for the histological subgroups and stage was calculated at fixed 43% specificity (0.266 cut-off). The classification rule identified with high sensitivity stage I NSCLC (94.7%) and stage II (100%), similarly to advanced stages III and IV (95.2 and 94.6%, respectively). The most prevalent NSCLC type, adenocarcinoma (ADC), also demonstrated a high sensitivity (93.2%), as in Squamous Cell Carcinoma (SqCC) and Large Cell Carcinoma (LCC) (100% both). All patients with SCLC were likewise detected with 100% sensitivity.

Among non-cancerous patients the panel correctly classified 41 out of 94 controls (43.6%), yielding a specificity of 40.9% for healthy and 46% for benign conditions of the lung.

Table 5 also includes the classification accuracy based on the results of the CT scan, specifically when no mass was detected. When additionally no nodules were found, the panel correctly classified all LC cases (7/7; 100% sensitivity). On the contrary, in the presence of nodules, our panel was able to classify 1 out of 3 controls (33.3% specificity).

## Discussion

Classification algorithms capable of guiding clinical decision-making constitute a valuable tool that can help predict LC, besides complement CT imaging^17^,^18^. In a previous work we described a three-marker panel for high-risk patients including the molecules EGF, sCD26 and CAL, and gender and age as confounders, and their implication in lung carcinogenesis was enclosed^19^,^20^,^21^,^22^,^23^. Here we provide an improved classification algorithm achieving a superior sensitivity for LC in the context of RDU. In this refined algorithm, besides the smoking status, the routinely used CEA was incorporated, corroborating its diagnostic capacity especially for late-stage tumours^4^,^24^,^25^,^26^. Briefly, our approach involves the measurement of EGF, sCD26, CAL and CEA to generate a classification score for each individual to predict LC.

As for colorectal and breast cancer, LC could also benefit from screening programs. However, at this time in Europe there are no LC screening recommendations though The European Society of Radiology and the European Respiratory Society recommend screening within a clinical trial or in routine clinical practice at certified medical centres^6^. Instead, the strategy implemented in many European hospitals to achieve an early detection is the acceleration in the time to diagnosis in the so called Rapid Diagnostic Units for LC^10^,^11^,^12^,^15^,^16^. Consequently, we intended to design a marker-based classification algorithm to be used in these Units, where the priority is to detect all LC cases (high sensitivity), in order to select those patients that should be immediately submitted to more invasive tests.

Individual analyses evidenced the usefulness of EGF, sCD26, CAL and CEA among the 8 molecules assayed, with AUCs between 0.698–0.759 for the training set and 0.716-0.871 for the validation set, headed in both cohorts by CAL. Among the four markers, differences were more frequent comparing NSCLC and controls, even at early stages as in the case of EGF and CAL. In relation to SCLC, sCD26 and CEA were the markers that better differentiated this histological group. The individual diagnostic potential of the four markers resulted in a modestly specific signature for the detection of LC when combined through a multivariate logistic Lasso regression approach that provided, by design, desirable sensitivity. This strategy demonstrated 97% sensitivity in the training set and for a >0.266 cut-off the classification algorithm showed a specificity of 43%. In the validation set sensitivity resulted in a fine 91.7% and 45.4% specificity. This modest specificity is of value in the clinical context of RDU with patients with respiratory symptoms and/or LC suspicion. Performance of our marker panel also outperforms that of a clinical model constituted by gender, age and smoking.

The classification accuracy including training and validation cohorts showed an overall sensitivity of 95.6% for LC. Among the 95% of NSCLC patients correctly classified, 94.7% of stage I tumours were detected. Regarding SCLC, the classification algorithm was effective for all the cases. Among controls, overall specificity resulted 43.6% and was not greatly affected by the nature of the controls themselves. Given the clinical dilemma of indeterminate nodules detected on CT-based screening due to elevated false positive rates^8^, we also evaluated our algorithm according to the absence/presence of nodules. All LC cases (100%) that had a negative CT-scan were correctly classified (6 out of 6 NSCLC and the SCLC case). On the contrary, among controls bearing nodules, our panel classified correctly 1 out of 3 patients. It should be noted that CT-scan data was available for all LC cases but only for 13.8% of controls (3 healthy and 10 benign cases), limiting the analysis.

In the last years several diagnostic multianalyte panels have been proposed for LC, with variable criteria for patient selection such as inclusion limited to NSCLC or absence of controls bearing benign pulmonary pathologies. Studies comparable to ours, at least with similar study population, are scarce. Molina *et al*.^25^ proposed a six marker panel (CEA, CA 15.3, Squamous Cell Carcinoma Antigen –SCC–, CYFRA 21.1, Neuron Specific Enolase –NSE– and Progastrin-releasing Peptide –ProGrp–) for patients with suspected LC based on the criterion of any of the markers elevated, proving a sensitivity of 88.5% and specificity of 82%, not validated.

Other studies document protein models combined with CT imaging techniques. Yang *et al*.^24^ reported for high-risk patients with no lesions on CT scan a panel, which resulted positive when at least one of the markers CEA, SCC, CYFRA 21.1 and Progastrin-releasing Peptide was altered, yielding a sensitivity of 76.6% and specificity of 94.4%, though they do not report data on another independent sample set. The algorithm established by Patz *et al*.^27^ based on the combination of nodule size and CEA, alpha-antitrypsin and SCC, rendered acceptable performance for classifying patients with indeterminate nodules (92% sensitivity and 74% specificity).

To date, only two blood tests based on marker panels have been translated into clinical or commercial setting. The EarlyCTD-Lung, which measures autoantibodies, was developed for the early detection of LC in high-risk population or as adjunct to CT^28^. Its performance was demonstrated in clinical practice, yielding 41% sensitivity and 87% specificity. The PAULA’s test (Protein Assays Using Lung cancer Analytes) is a 4-marker panel comprising three tumour antigens (CEA, CA125 and CYFRA 21.1) and one autoantibody (NY-ESO1), intended for early NSCLC tumours in high-risk patients. In a validation set the panel discriminated NSCLC (with 67% early-stage) from healthy controls with a sensitivity of 77% and specificity of 80%. However its clinical applicability is limited since benign conditions were not included^4^. None of the cited studies pursued such a high sensitivity as we do, which would probably derive in a diminished specificity. In these circumstances, we would affirm the promising value of our 4-marker panel.

Our model building procedure is based on regularized regression models which are intended to be more flexible and resistant to overfitting compared to stepwise approaches^29^,^30^,^31^. Furthermore, by design, our method identifies models which guarantee the optimization of the derived classification rule by choosing the penalty parameter and cut-off which maximizes specificity, assuring a predefined sensitivity. The adaptive nature of our method constitutes one of the strengths of our study. Alternative model building techniques established by first choosing a logistic model based on a reduced set of variables by minimizing the AIC or BIC, and then determining a cut-off depending on the given classification setting, are less flexible, and in general our method outperforms these approaches since it is specifically designed for optimizing classification performance. Moreover, approaches based on exhaustive evaluation of all possible sub-models become rapidly unfeasible when increasing the number of candidate markers, whereas our approach, since it relies on shrinkage, is expected to perform well in such situations.

In Supplementary Table S4 we have included two logistic regression models (two-stage) and derived classification rules for selected 90% and 95% sensitivities. As observed, the obtained models and classification rules are not uniformly optimal and their performance varies according to the classification situation. For example, the classification rule based on BIC performs well for the cut-off that provides 95% sensitivity, while its performance is considerably worse for the cut-off corresponding to 90% sensitivity. Alternatively, if we focus on the AIC as optimal criterion, this method outperforms alternative Lasso-based and BIC for 90% sensitivity, but it presents an inferior performance when we focus on higher sensitivities.

Given the complex challenge of developing an optimal diagnostic panel for LC, a proper study design is also of crucial importance. Besides the consciousness in the statistical approach aforementioned, the inclusion of both benign and healthy individuals in the control group, as well as the two main histological tumours (NSCLC and SCLC) is a strong point of our study. Another important feature is that samples from all the individuals were prospectively collected at their first visit to the Pneumology Service in the presence of respiratory symptoms, reflecting the clinical setting of a RDU. For the refinement of the diagnostic algorithm we have also included information related to tobacco, which constitutes a well-established risk factor^32^ and is usually not contemplated in studies developing diagnostic panels.

One of the advantages of our classification algorithm is that only 4 molecules comprise the panel, and 2 of them are already established in hospitals: CEA is routinely measured for various types of tumours, while CAL is also quantified for its utility in inflammatory colon processes^33^. This makes our 4-marker panel simple and affordable to guide clinical decision-making and complement CT scan. Additionally, we are currently working on an interactive web application to facilitate the implementation of the classification algorithm in the biomedical community, based on the Shiny web application for R^34^.

Regarding the limitations of the study, the number of patients was modest for both training and validation sets, particularly for SCLC cases. A possible shortcoming could be the lack of information related to tobacco consumption, which perhaps could have contributed to the improvement of the diagnostic algorithm.

In summary, we defined a modestly specific 4-marker classification algorithm that provided, by design, desirable sensitivity for the detection of LC, conceived to be useful among symptomatic high-risk individuals derived to LC-RDU. The next step along the complicated road to reach the clinical implementation is the validation of our panel in a large, multi-centric cohort.

## Methods

### Study Population

Between May 2007 and January 2011, 186 patients with respiratory symptoms were prospectively recruited at the Pneumology Service of Hospital Álvaro Cunqueiro EOXI Vigo (Spain). The study population included patients finally diagnosed of LC, and a control cohort with subjects diagnosed of benign lung disease and healthy subjects with no respiratory pathology. Exclusion criteria included relapse or progression of a cancer previously diagnosed, and chemo-or radiotherapy treatment.

Clinical guidelines from the American College of Chest Physicians were followed for LC diagnosis^13^,^14^. Histological assessment of tumours followed the WHO criteria^35^ and staging was performed according to the 7^th^ edition of TNM^36^.

Recruited individuals were divided into a training set for panel development, and in another set for validation of the algorithm. The training set consisted of 140 individuals and included 68 LC cases (80.9% men, median age 69.5 years). The control cohort included 72 subjects with a median age of 61 years and 63.6% males. The validation set consisted of 46 individuals (24 LC and 22 controls). Patient demographics are outlined in Supplementary Table S5.

The study followed the clinical-ethical practices of the Spanish Government and the Helsinki Declaration, and was approved by the Galician Ethical Committee for Clinical Research. All patients provided written informed consent.

### Determination of Markers Concentration

Blood samples were collected from all patients at their first visit to the Service, when bronchoscopy was performed. Serum was obtained and stored at −20 °C until analysis.

Measurement of EGF (R&D Systems, Minneapolis, USA), sCD26 (eBioscience, Wien, Austria) and Calprotectin (Hycult Biotechnology, Uden, the Netherlands) concentrations were conducted using enzyme-linked immunosorbent assays (ELISA). Absorbance readings were collected on an EnVision Multilabel Plate Reader (Perkin Elmer).

To measure the amount of serum MMPs, CEA and CYFRA 21.1 multiplexed bead-based immunoassays were used. Levels of MMP-1, MMP-7 and MMP-9 were determined with the Human MMP Panel 2 Magnetic Bead Kit, while CEA and CYFRA 21.1 were part of the Circulating Cancer Biomarker Magnetic Bead Panel 1 (EMD Millipore, Missouri, USA). Fluorescence was read on a Luminex 200™ with BioPlex Manager™ software (Bio-Rad, Hercules, CA), using a 5-parameter logistic fitting for deriving protein concentration in samples.

### Statistical Methods

#### Individual Marker Analysis

Non-parametric Mann-Whitney U test was used for two-sample group comparisons of continuous variables, while Fisher test was applied for comparison of qualitative variables. Benjamini-Hochberg method for controlling the false discovery rate^37^ was used to correct *P*-values for multiple group comparisons. Linear regression models were used to study markers’ association with LC presence, histology and stage adjusted for the risk factors gender, age and smoking. The discriminatory ability of markers for LC was evaluated by Receiver Operating Characteristics Curve (ROC) providing the Area Under the Curve (AUC). All tests were two-sided and *P*-values ≤ 0.05 were considered statistically significant. Statistical software SPSS 22.0 (SPSS Inc., Chicago, IL) and R program package (Wirtschafts Universität, Wien, Austria) were used to perform these analyses.

### Marker Panel Selection and Classification Algorithm Development

Marker concentrations were log_10_-transformed before multivariate analysis to reduce the skewness. We derived a classification rule based on a multivariate combination of the studied markers based on logistic Lasso regression^38^ fitted in the training set and including age, gender and smoking as fixed effects. This procedure was also used to obtain a clinical model in which only the variables age, gender and smoking were included. Lasso regularization imposes a penalization over the maximum likelihood estimates of the usual regression coefficients so that they are shrunk towards zero. Actually, some of the resulting coefficients can be exactly zero, and hence Lasso shrinkage performs automatic variable selection. The optimal amount of shrinkage is controlled by the selection of the penalization parameter which maximizes the out-of-sample performance (in terms of some pre-defined criterion) of the model. In our algorithm, we simultaneously chose the penalty parameter and cut-off point which provides the classification rule with maximum specificity, given a fixed value of sensitivity (95%). Namely, we use 10-fold cross validation in the training set and for each possible value of the penalty parameter we apply the resulting estimated coefficients to the out-of-sample data, obtaining case probability scores for each observation of the training set. Each of these scores was subsequently dichotomized to guarantee the desired level of sensitivity. Finally, we choose the penalty parameter which maximized the specificity. Further details concerning the Lasso procedure and the proposed algorithm are displayed as Supplementary Material S1.

For prediction of a new individual’s diagnosis, the selected classification rule was applied. Based on the coefficients of the regression model, the classification algorithm calculates for a new patient a single score based on the estimated predicted probabilities (*p*) of presenting lung cancer as a function of markers concentrations and demographic variables. A new individual will be classified as cancer if *p* is higher than the cut-off estimated in the training set, while classified as non-cancer when the resulting score is below the cut-off.

Applying the Lasso regression model to the train and test samples, their probability scores were obtained and the diagnostic performance of the classification rule was evaluated by providing sensitivity, specificity and predictive values. ROC curves were elaborated for both the Lasso-based marker model and clinical model, providing the AUC. DeLong test was applied for comparison of AUC values of these models^39^.

For sake of comparison, we evaluated the performance of two alternative two-stage methods based on first selecting an optimal logistic model, based, respectively, on exhaustive sub-model evaluation and selection based on minimization of Akaike information criterion (AIC)^40^ and Bayesian information criterion (BIC)^41^ and secondly, determining the optimal cut-off for guarantying the desired level of sensitivity.

All multivariate calculations were performed using the R program package (Wirtschafts Universität, Wien, Austria).

## Additional Information

**How to cite this article**: Blanco-Prieto, S. *et al*. Highly Sensitive Marker Panel for Guidance in Lung Cancer Rapid Diagnostic Units. *Sci. Rep.* **7**, 41151; doi: 10.1038/srep41151 (2017).

**Publisher's note:** Springer Nature remains neutral with regard to jurisdictional claims in published maps and institutional affiliations.

## Supplementary Material

Supplementary Information

## Figures and Tables

**Table 1:** Markers Selected for the Development of a Diagnostic Panel for Lung Cancer.

Marker	Function in Cancer	Usefulness in LC diagnosis
Epidermal Growth Factor (EGF)	Binding of EGF to receptor promotes tumour growth and progression^42^	Suitable discrimination of LC/NSCLC from healthy and benign lung pathologies^19^,^20^
sCD26	Immune regulation and co-stimulatory activities^43^ Tumour suppressor protein in NSCLC^44^	Suitable diagnostic potential for LC vs healthy^21^, healthy/ benign^19^ or NSCLC-MPE/NSCLC-PMPE^22^
Calprotectin (CAL)	Antimicrobial and pro-inflammatory functions, tumour development^45^ Mediate lung metastases^46^	Promising marker in LC derived by pleural effusion^23^ and LC^19^
Matrix Metalloproteinases (MMP-1, −7, −9)	Extracellular matrix degradation leading to cancer invasion and metastasis, processing of growth factors, regulation of apoptosis and angiogenesis, tumour-associated inflammation and immune escape^47^	MMP-1: Poor diagnostic capacity for LC vs healthy controls in plasma^48^
		MMP-7: Moderate diagnostic potential for NSCLC vs healthy and benign lung disease^49^
		MMP-9: Good diagnostic potential for LC vs control and benign lung affections^50^,^51^
Carcinoembryonic Antigen (CEA)	Belongs to immunoglobulin superfamily, acting in cell adhesion and innate immunity^52^	Moderate diagnostic potential for LC vs non-malignant pathologies^4^,^24^,^25^
CYFRA 21.1	Fragment of Cytokeratin 19, constituent of cytoskeleton and expressed in epithelial differentiation^53^	Moderate diagnostic potential for LC vs non-malignant pathologies^4^,^24^,^25^

Abbreviations: LC = Lung Cancer, NSCLC = Non Small Cell Lung Cancer, MPE = Malignant Pleural Effusion, PMPE = paramalignant pleural effusion.

**Table 2:** Serum Markers in Lung Cancer and Controls in the Training Set.

Marker	Control/Case^a^	Median	Range	*P*^b^	Adjusted effect (95% CI)^c^	AUC (95% CI)
**EGF**	**Control**	**336.86**	**40.13–1187.06**			
(pg/mL)	Healthy	247.60	40.13–972.90			
	Benign	419.67	43.25–1187.06			
	**LC**	**571.70**	**40.75–1716.30**	**0.001**	0.238 (0.140–0,336)*	0.698 (0.615–0.773)
**sCD26**	**Control**	**471.50**	**122.00–1092.00**			
(ng/mL)	Healthy	509.00	237.00–886.00			
	Benign	431.50	122.00–1092.00			
	**LC**	**356.00**	**136.00–1192.00**	**0.001**	−0.097 (−0.148–^−^0.047)*	0.711 (0.629–0.785)
**CAL**	**Control**	**127.87**	**7.56–421.23**			
(ng/mL)	Healthy	105.47	7.56–362.29			
	Benign	158.13	33.13–421.23			
	**LC**	**221.21**	**48.33–438.32**	**0.001**	0.263 (0.190–0.336)*	0.759 (0.679-0.827)
**MMP-1**	**Control**	**5459.61**	**1186.61–23960.37**			
(pg/mL)	Healthy	4664.94	1186.61–23635.33			
	Benign	6420.51	1207.70–23960.37			
	**LC**	**7133.87**	**1450.34–41668.33**	**0.047**	0.089 (−0.008–0.187)	0.597 (0.511-0.679)
**MMP-7**	**Control**	**21761.35**	**5026.14–79977.27**			
(pg/mL)	Healthy	21237.20	10383.69–60968.91			
	Benign	22286.74	5026.14–79977.27			
	**LC**	**26710.65**	**5383.18–79809.13**	**0.013**	0.020 (−0.040–0.081)	0.624 (0.539-0.705)
**MMP-9**	**Control**	**177.60**	**52.79–3611.59**			
(ng/mL)	Healthy	177.19	52.79–743.25			
	Benign	181.74	57.41–3611.59			
	**LC**	**311.86**	**21.06–1914.00**	**0.001**	0.270 (0.167–0.373)*	0.729 (0.648–0.801)
**CEA**	**Control**	**837.26**	**170.84–4070.71**			
(pg/mL)	Healthy	929.50	171.03–4070.71			
	Benign	559.51	170.84–2828.15			
	**LC**	**2051.64**	**141.16–136039.19**	**0.001**	0.494 (0.333–0.656)*	0.744 (0.663–0.814)
**CYFRA 21.1**	**Control**	**227.05**	**0.00–19314.33**			
(pg/mL)	Healthy	0.00	0.00–16592.63			
	Benign	1052.47	0.00–19314.33			
	**LC**	**3007.09**	**0.00–173410.17**	**0.001**	1.080 (0.587–1.573)*	0.734 (0.653–0.805)

Abbreviations: LC = Lung Cancer.

^a^Sample size in training set: Control n = 72 (Healthy n = 36, Benign n = 36), LC n = 68.

^b^Mann-Whitney U test for comparison between the cancer and control group corrected by Benjamini-Hochberg method to control familywise error under multiple comparisons.

^c^Adjusted effects and 95% confidence intervals of the case/control status on each of the log-transformed markers considered as outcome in lineal regression model adjusted for gender, age and smoking. *P-value < 0.001.

**Table 3:** Distribution of Serum Markers in Lung Cancer by Histology and Stage and Comparison with Controls in the Training Set.

Marker	Control/Case^a^	Median	Range	*P*^b^	Adjusted effect (95% CI)^c^
**EGF**	**Control**	**336.86**	**40.13–1187.06**		
(pg/mL)	**NSCLC**	**601.97**	**144.23–1176.15**	**0.002**	0.277 (0.168–0.386)*
	Early (I + II)	785.28	388.23–1159.73	0.002	0.409 (0.236–0.581)*
	Late (III + IV)	528.51	144.23–1176.15	0.002	0.231 (0.109–0.354)*
	**SCLC**	**295.48**	**40.75–1716.30**	**0.798**	**−0.039 (−0.293–0.216)**
	Limited	55.97	40.75–201.63	—	
	Extended	440.64	264.30–1716.30	—	
**sCD26**	**Control**	**471.50**	**122.00–1092.00**		
(ng/mL)	**NSCLC**	**356.00**	**136.00–945.00**	**0.002**	**−0.095 (−0.153–** ^**−**^0.038)*
	Early (I + II)	432.00	206.00–640.00	0.235	-0.032 (−0.114–0.049)
	Late (III + IV)	341.00	136.00–945.00	0.002	−0.116 (−0.179– ^−^0.054)*
	**SCLC**	**294.00**	**208.00–1092.00**	**0.040**	**−0.073 (−0.184–0.037)**
	Limited	370.00	339.00–1192.00	—	
	Extended	288.00	208.00–541.00	—	
**CAL**	**Control**	**127.87**	**7.56–421.23**		
(ng/mL)	**NSCLC**	**221.06**	**87.19–438.32**	**0.002**	0.250 (0.160–0.341)*
	Early (I + II)	193.72	120.73–340.67	0.017	0.188 (0.033–0.343)*
	Late (III + IV)	238.59	87.19–438.32	0.002	0.275 (0.172–0.378)*
	**SCLC**	**245.69**	**48.33–422.20**	**0.112**	**0.203 (−0.014–0.419)**
	Limited	97.32	91.06–422.20	—	
	Extended	279.34	48.33–355.19	—	
**MMP-1**	**Control**	**5459.61**	**1186.61–23960.37**		
(pg/mL)	**NSCLC**	**7132.52**	**1450.34–41668.33**	**0.093**	**0.071 (−0.049–0.192)**
	Early (I + II)	7800.42	1784.41–30180.33	0.235	0.087 (-0.106-0.279)
	Late (III + IV)	7132.52	1450.34–41668.33	0.131	0.066 (−0.065–0.198)
	**SCLC**	**7135.23**	**2220.44–18900.42**	**0.279**	**0.083 (−0.158–0.323)**
	Limited	7135.23	4890.35–18900.42	—	
	Extended	8114.52	2220.44–14246.29	—	
**MMP-7**	**Control**	**21761.35**	**5026.14–79977.27**		
(pg/mL)	**NSCLC**	**26485.45**	**5383.18–79809.13**	**0.048**	**0.021 (−0.052–0.095)**
	Early (I + II)	24615.56	8551.00–51000.43	0.726	−0.031 (−0.136–0.074)
	Late (III + IV)	26680.26	5383.18–79809.13	0.023	0.039 (−0.041–0.118)
	**SCLC**	**28177.70**	**8231.19–43429.13**	**0.056**	**0.052 (−0.082–0.186)**
	Limited	26874.44	26710.65–40252.39	—	
	Extended	30110.14	8231.19–43429.13	—	
**MMP-9**	**Control**	**177.60**	**52.79–3611.59**		
(ng/mL)	**NSCLC**	**340.19**	**21.06–1914.00**	**0.002**	0.257 (0.135–0.379)*
	Early (I + II)	379.94	174.70–1688.00	0.002	0.285 (0.101–0.470)*
	Late (III + IV)	299.65	21.06–1914.00	0.002	0.244 (0.106–0.382)*
	**SCLC**	**225.61**	**65.58–786.34**	**0.798**	**0.013 (−0.235–0.262)**
	Limited	115.93	65.58–253.19	—	
	Extended	298.21	76.78–786.34	—	
**CEA**	**Control**	**837.26**	**170.84–4070.71**		
(pg/mL)	**NSCLC**	**1783.93**	**141.16–136039.19**	**0.002**	0.467 (0.266–0.668)*
	Early (I + II)	1093.08	353.26–21684.85	0.140	0.154 (−0.050–0.358)
	Late (III + IV)	2750.49	141.16–136039.19	0.002	0.567 (0.348–0.787)*
	**SCLC**	**3704.96**	**1147.58–82300.26**	**0.002**	0.818 (0.541–1.094)*
	Limited	2092.47	1147.58–82300.26	—	
	Extended	3884.15	1667.40–29844.72	—	
**CYFRA 21.1**	**Control**	**227.05**	**0.00–19314.33**		
(pg/mL)	**NSCLC**	**2910.66**	**0.00–173410.17**	**0.002**	0.939 (0.348–1.531)*
	Early (I + II)	1181.22	0.00–7309.16	0.104	0.503 (−0.382–1.387)
	Late (III + IV)	4791.34	0.00–173410.17	0.002	1.105 (0.459–1.752)*
	**SCLC**	**5886.75**	**0.00–12228.10**	**0.012**	1.285 (0.154–2.415)*
	Limited	1754.30	219.64–6610.22	—	
	Extended	5965.40	0.00-12228.10	—	

Abbreviations: NSCLC = Non-Small Cell Lung Cancer, SCLC = Small Cell Lung Cancer.

^a^Sample size in training set: Control n = 72, NSCLC n = 59 (Early stage n = 16, Late stage n = 43), SCLC n = 9 (Limited stage n = 3, Extended stage n = 6).

^b^Mann-Whitney U test for comparison between the control groups and lung cancer stratified by histology and stage corrected by Benjamini-Hochberg method to control familywise error under multiple comparisons.

^c^Adjusted effects and 95% confidence intervals of histology and stage on each of the log-transformed markers considered as outcome in lineal regression model adjusted for gender, age and smoking. *P-value statistically significant.

**Table 4:** Performance of the Four-Marker Panel and EGF, sCD26, CAL and CEA in the Diagnosis of Lung Cancer.

Training Set	Cut-off	Sn (%)	Sp (%)	PPV^a^ (%)	NPV^a^ (%)	AUC (95% CI)^b^
Multivariate Algorithm: EGF, sCD26, CAL, CEA	>0.266	97	43	57.6	94.7	0.873 (0.811–0.925)
EGF	>178.48 pg/mL	95	22.2	49.4	84.8	0.698 (0.615–0.773)
sCD26	≤637.2 ng/mL	95	13.9	46.8	77.7	0.711(0.629–0.785)
CAL	>96.37 ng/mL	95	30.6	52.2	88.4	0.759 (0.679–0.827)
CEA	>258.2 pg/mL	95	11.1	46	73.6	0.744 (0.663–0.814)
Clinical Model^c^	>0.237	95	26.4	50.8	86.9	0.717 (0.637-0.799)
**Validation Set**						
Multivariate Algorithm: EGF, sCD26, CAL, CEA	>0.266	91.7	45.4	57.3	87.3	0.837 (0.718-0.936)
Clinical Model^c^	>0.237	91.7	27.3	50.2	80.5	0.659 (0.488-0.816)

Abbreviations: Sn = Sensitivity, Sp = Specificity, PPV = Predictive Positive Value, NPV = Negative Predictive Value.

^a^Positive and negative predictive values were estimated assuming a prevalence of lung cancer of 44.4% (QDU of the Pneumology Service of Hospital Álvaro Cunqueiro EOXI Vigo).

^b^AUC and 95% CI evaluated in the training test is not protected against overfitting.

^c^Clinical model includes gender, age and smoking.

**Table 5:** Classification Accuracy of the Multivariate Algorithm for Subgroups of Patients in the Combined Set (Training and Validation Set).

		Cases correctly classified^a^/Total cases	% Sn at 43% Sp
**Lung Cancer**		88/92	95.6
NSCLC		76/80	95
	I	18/19	94.7
	II	3/3	100
	III	20/21	95.2
	IV	35/37	94.6
	ADC	41/44	93.2
	SqCC	20/20	100
	LCC	13/13	100
	BAC	1/2	50
	ND	1/1	100
SCLC		12/12	100
		**Cases correctly classified**^**a**^**/Total cases**	**% Sp at 95% Sn**
**Control**		41/94	43.6
Healthy		18/44	40.9
Benign		23/50	46
	RI	19/41	46.3
	ILD	4/9	44.4
**Accuracy in the Classification of Cancer and Controls regarding CT imaging**			
**Absence of Nodules in CT scan**		**Cases correctly classified**^**a**^**/Total cases**	**% Sn at 43% Sp**
**Lung Cancer**		7/7	100
NSCLC		6/6	100
	I	1/1	100
	II	2/2	100
	III	1/1	100
	IV	2/2	100
SCLC	Extended	1/1	100
**Presence of Nodules in CT scan**		**Cases correctly classified**^**a**^**/Total cases**	**% Sp at 95% Sn**
**Control**		1/3	33.3
Healthy		0/1	0
Benign		1/2	50

Abbreviations: Sn = Sensitivity, Sp = Specificity, NSCLC = Non Small Cell Lung Cancer, ADC = Adenocarcinoma, SqCC = Squamous Cell Carcinoma, LCC = Large Cell Carcinoma, BAC = Bronchioloalveolar Carcinoma, ND = Not Differentiated Carcinoma, SCLC = Small Cell Lung Cancer, RI = Respiratory Infection, ILD = Interstitial Lung Disease.

^a^Cut-off *p* = 0.266 for a sensitivity of 97% and specificity of 43% in the training set.
