# Supplementary material for: Highly Sensitive Marker Panel for Guidance in Lung Cancer Rapid Diagnostic Units
Source: Sci Rep. 2017 Jan 24;7:41151. doi: 10.1038/srep41151 (PMC5259733; doi:10.1038/srep41151)
Supplement: Supplementary Information [file srep41151-s1.pdf]

Title: **Highly Sensitive Marker Panel for Guidance in Lung Cancer Rapid Diagnostic Units**

Authors: Sonia Blanco-Prieto, Loretta De Chiara, Mar Rodríguez-Girondo, Lorena Vázquez-Iglesias, Francisco Javier Rodríguez-Berrocal, Alberto Fernández-Villar, María Isabel Botana-Rial, María Páez de la Cadena

SUPPLEMENTARY FIGURE S1: Annotated Heatmap based on Pearson Correlation of the Studied Molecular Markers in the Training Set

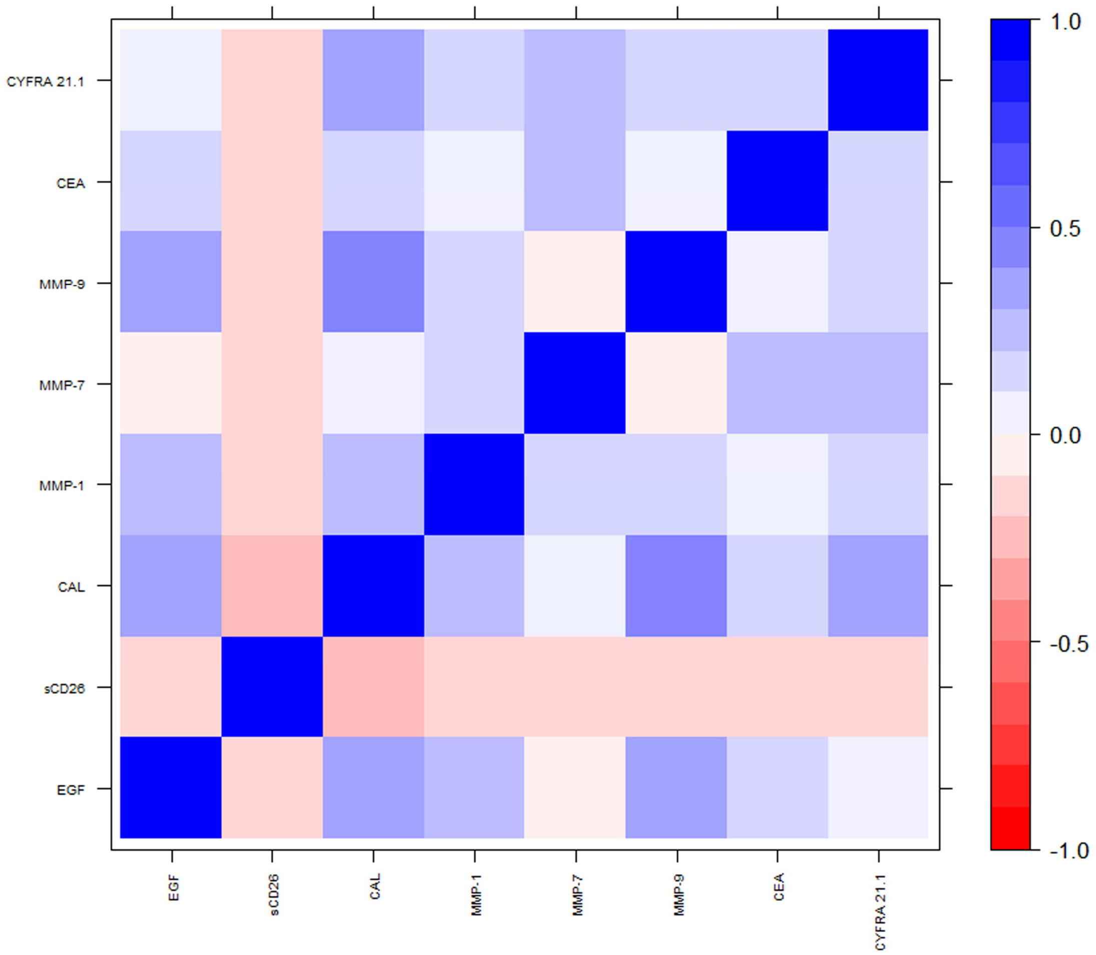

SUPPLEMENTARY TABLE S1. Association of Markers with Gender, Age and Smoking in the Training Set

Marker	Gender[a]			Age[a]			Smoking[a]		
	**Male** (n=101)	**Female** (n=39)	***P*[b]**	**≤65 years** (n=73)	**>65 years** (n=67)	***P*[b]**	**Yes** (n=112)	**No** (n=28)	***P*[b]**
**EGF** (pg/mL)	438.04<br>40.13-1716.30	347.10<br>116.45-1187.06	0.490	433.91<br>40.75-1176.15	431.55<br>40.13-1716.30	0.995	465.81<br>40.13-1716.30	334.20<br>98.01-727.55	0.028
**sCD26** (ng/mL)	380.00<br>136.00-1192.00	453.00<br>122.00-945.00	0.018	470.00<br>159.00-1092.00	361.00<br>122.00-1192.00	0.001	383.50<br>136.00-1192.00	458.00<br>122.00-102.00	0.125
**CAL** (ng/mL)	181.44<br>7.56-438.32	199.22<br>33.13-430.40	0.831	181.48<br>7.56-438.32	190.39<br>33.13-430.40	0.501	190.84<br>7.56-438.32	158.14<br>33.13-430.40	0.300
**MMP-1** (pg/mL)	6060.28<br>1207.70-41668.33	5862.96<br>1186.61-22436.23	0.258	5988.68<br>1186.61-22595.70	5916.00<br>1207.70-41668.33	0.483	6061.80<br>1186.61-41668.33	5317.19<br>1207.70-22595.70	0.105
**MMP-7** (pg/mL)	24324.18<br>5026.14-79977.27	22443.39<br>5383.18-50903.24	0.285	21936.90<br>5026.14-53466.87	27755.14<br>5383.18-79977.27	0.001	25145.77<br>5026.14-79977.27	20767.69<br>5383.18-50903.24	0.026
**MMP-9** (ng/mL)	261.66<br>21.06-3611.59	215.34<br>52.79-3300.50	0.154	224.96<br>21.06-3611.59	261.66<br>52.79-1526.50	0.783	261.63<br>21.06-3611.59	183.55<br>52.79-3300.50	0.077
**CEA** (pg/mL)	1261.73<br>141.16-136039.19	1050.94<br>187.02-82300.26	0.478	1007.82<br>161.95-82300.26	1458.80<br>141.16-136039.19	0.094	1356.80<br>141.16-136039.19	828.39<br>170.84-102098.59	0.061
**CYFRA 21.1** (pg/mL)	1250.35<br>0.00-173410.17	475.86<br>0.00-35365.75	0.096	446.05<br>0.00-43641.44	1932.84<br>0.00-173410.17	0.004	1155.57<br>0.00-173410.17	500.10<br>0.00-19314.33	0.202

[a] Median and range values provided
[b] Mann-Whitney U test

SUPPLEMENTARY MATERIAL S1: Details of Classification Algorithm based on Lasso Logistic Regression.

We derived a classification rule based on a multivariate combination of the studied markers based on logistic Lasso regression[1]. The general aim is to build a decision rule to predict a binary outcome **y** in terms of a set of p (molecular) markers **X**=(X_1,…,X_p), using a training sample of size *n*. For each observation i, we estimate its class membership as $\hat{y}_i = I(\hat{p}_i > \hat{c}), \quad \hat{p}_i = \hat{P}(y_i = 1|x_{1i}, \dots, x_{pi})$, where $\hat{p}_i$ are the estimated membership probabilities and $\hat{c}$ is the estimated optimal cut-off point based on $\hat{p}_i$ and a given optimality criterion. We consider a logistic lasso regression for the simultaneous estimation of $\hat{p}_i$ and $\hat{c}$ in terms of the set of predictors $\boldsymbol{X}$, through the estimation of the regression coefficients $\boldsymbol{\beta} = (\beta_1, \dots, \beta_p)$ corresponding to each of the p considered markers. Specifically, $logit(\hat{p}_i) = log\left(\frac{\hat{p}_i}{1-\hat{p}_i}\right) = \beta_0 + x_{i1}\beta_1 + \cdots + x_{ip}\beta_{1p}$ and the estimation of $\boldsymbol{\beta} = (\beta_1, \dots, \beta_p)$ is conducted by maximizing the penalized log-likelihood

$$\sum_{i=1}^{n}[y_i \log(\hat{p}_i) + (1 - y_i)\log(1 - \hat{p}_i)] - \Lambda pen(\boldsymbol{\beta})$$

The penalty parameter Λ regularizes the traditional maximum likelihood coefficients by shrinking large coefficients in order to control the bias-variance trade-off. We use a Lasso-type [1] penalty, with $pen(\boldsymbol{\beta}) = ||\boldsymbol{\beta}||_1 = \sum_{j=1}^{p}|\beta_j|$, which allows for variable selection since some of the resulting coefficients can be exactly zero. In practice, the final estimated regression coefficients $\boldsymbol{\beta}$ are determined by the choice of the optimal (in some sense) Λ, Λ_{opt}.

In our algorithm, we simultaneously chose the penalty parameter Λ_{opt} and cut-off point $\hat{c}$ which provide the classification rule with maximum specificity, given a fixed value of

sensitivity equal to 95%, using 10-fold cross validation in the training set. For each possible value of the penalty parameter (we considered a grid of 170 values of Λ from 0.001 to 0.17), we obtain the corresponding set of regression coefficients in each of the 10 partitions of the training set (leaving aside 1/10 of the training set at each time), and we apply the resulting estimated coefficients to the out-of-sample data, obtaining case probability scores $\hat{p}_i^{\Lambda}$ for each observation of the training set and possible value of Λ. Each of these 170 scores were subsequently dichotomized to guarantee the desired level of sensitivity 95 %, providing $\hat{c}_1^{\Lambda 1}$,..., $\hat{c}_1^{\Lambda 170}$ as possible optimal cut-off points, with different level of specificity. Finally, we chose the penalty parameter Λ_{opt} whose corresponding $\hat{c}_1^{\Lambda opt}$ maximized the specificity.

The algorithm was implemented using the R program (Wirtschafts Universität, Wien, Austria) and using the package *glmnet* [2] for Lasso regularization.

In our case, we considered 8 molecular markers and three extra clinical markers (age, gender and smoking) that entered the model without penalization ($pen(\boldsymbol{\beta}) = ||\boldsymbol{\beta}||_1 = \sum_{j=1}^{p}|\beta_j|$ does not include the regression coefficients corresponding to age, gender and smoking).

The final classification rule corresponds to $\Lambda_{opt} = 0.059$ and $\hat{c} = 0.266$, i.e. $\hat{y}_i = I(\hat{p}_i > 0.266)$, where $\hat{p}_i$ is given by:

$$logit(\hat{p}) = -12.362 + 1.735 log_{10}CAL + 0.796 log_{10}CEA - 0.067 log_{10}CD26 + 0.405 log_{10}EGF + 0.035 age - 0.250 I(gender = woman) + 1.715 I(smoking)$$

REFERENCES:

1. Tibshirani, R. Regression shrinkage and selection via the lasso. *J. Royal Statist. Soc. B*. **58,** 267-288 (1996).

2. Friedman, J., Hastie, T. & Tibshirani, R. Regularization Paths for Generalized Linear Models via Coordinate Descent. *Journal of Statistical Software*. **33**, 1-22 (2010).

SUPPLEMENTARY FIGURE S2: Optimal Lasso Penalization Parameter.

Specificities corresponding to a fixed value of 95% sensitivity plotted for each possible value of the penalization parameter Λ in logistic Lasso regression. Dashed lines indicate the optimal selected penalization parameter (Λ_{opt}) corresponding to the maximum specificity, determined by 10-fold cross-validation.

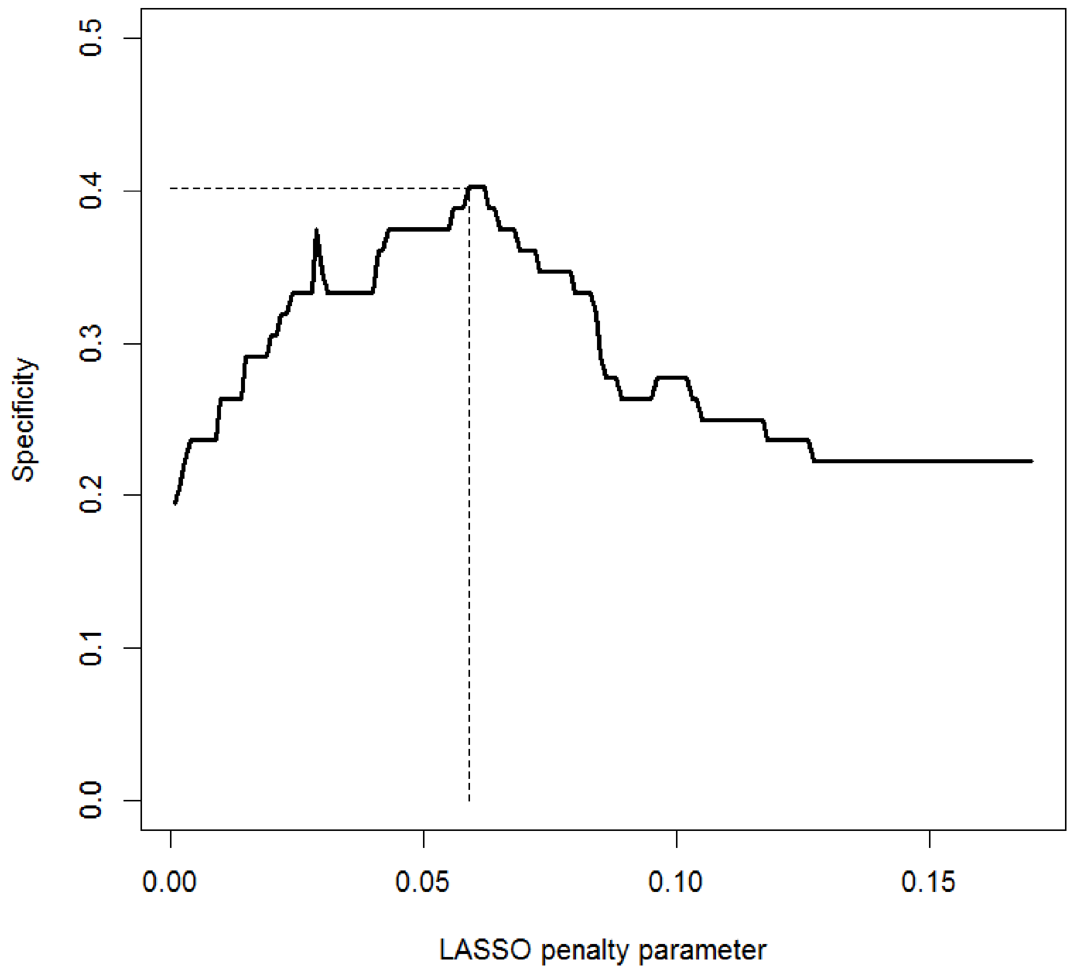

SUPPLEMENTARY TABLE S2: Diagnostic Measurements of the Proposed 4-Marker Model

Criterion	Measurement
Deviance	131.36
AIC	145.36
BIC	165.95

Abbreviations: AIC= Akaike Information Criterion, BIC=Bayesian Information Criterion

SUPPLEMENTARY TABLE S3: Levels of the Serum Markers included in the 4-Marker Panel for the Validation Set

Marker	Case/Control[a]	Median	Range	P^b	AUC (95% CI)
**EGF** (pg/mL)	**Control**	**349.69**	**62.63-1160.42**		
	Healthy	301.65	109.37-519.29		
	Benign	454.32	62.63-1160.42		
	**LC**	**759.18**	**78.37-1375.50**	**0.008**	0.727 (0.576-0.848)
	NSCLC I+II	839.81	722.66-1176.89	0.001	
	NSCLC III+IV	574.51	170.22-1375.50	0.061	
	**SCLC**	**585.35**	**78.37-776.83**	**0.783**	
	SCLC Limited	427.60	78.37-776.83	-	
	SCLC Extended	585.35	-	-	
**sCD26** (ng/mL)	**Control**	**456.00**	**228.00-1025.00**		
	Healthy	605.50	308.00-1025.00		
	Benign	434.00	228.00-998.00		
	**LC**	**380.50**	**165.00-846.00**	**0.012**	0.716 (0.564-0.839)
	NSCLC I+II	409.00	250.00-598.00	0.214	
	NSCLC III+IV	365.00	165.00-778.00	0.014	
	**SCLC**	**306.00**	**249.00-846.00**	**0.353**	
	SCLC Limited	547.50	249.00-846.00	-	
	SCLC Extended	306.00	-	-	
**CAL** (ng/mL)	**Control**	**117.94**	**38.67-247.36**		
	Healthy	117.94	39.16-247.36		
	Benign	128.61	38.67-234.44		
	**LC**	**258.33**	**111.69-482.89**	**<0.001**	0.871 (0.739-0.952)
	NSCLC I+II	265.78	154.34-426.99	0.007	
	NSCLC III+IV	261.10	126.50-482.89	<0.001	
	**SCLC**	**190.52**	**111.69-374.88**	**0.238**	
	SCLC Limited	243.28	111.69-374.88	-	
	SCLC Extended	190.52	-	-	
**CEA** (pg/mL)	**Control**	**764.77**	**236.07-4616.82**		
	Healthy	764.77	236.07-4203.63		
	Benign	788.36	354.97-4616.82		
	**LC**	**2102.93**	**374.15-42679.99**	**0.003**	0.759 (0.611-0.873)
	NSCLC I+II	1787.12	839.29-5201.68	0.039	
	NSCLC III+IV	2284.68	374.15-42679.99	0.022	
	**SCLC**	**5060.04**	**1097.96-10139.27**	**0.027**	
	SCLC Limited	3079.00	1097.96-5060.04	-	
	SCLC Extended	10139.27	-	-	

Abbreviations: LC=Lung Cancer, NSCLC=Non-Small Cell Lung Cancer, SCLC=Small Cell Lung Cancer

[a] Sample size in validation set: Control n=22 (Healthy n=8, Benign n=14), NSCLC n=21 (Early stage I+II n=6, Late stage III+IV n=15), SCLC n=3 (Limited stage n=2, Extended stage n=1)

[b]Mann-Whitney U test for the comparison between the cancer and control groups, and comparison between NSCLC stratified by early and advanced stage *versus* controls

SUPPLEMENTARY TABLE S4: Alternative Model Building Procedures based on AIC and BIC Criteria

		Minimize AIC[a]		Minimize BIC[a]	
		Sn=95%	Sn=90%	Sn=95%	Sn=90%
**Markers included**		EGF, sCD26, CAL, CEA, CYFRA 21.1		EGF, CAL, CEA,	
**Deviance**		107.86		113.50	
**AIC**		125.86		127.45	
**BIC**		152.34		148.09	
**Cut-off 95% Sn**		>0.057		>0.141	
	Sn, Sp (%) train	95.6, 33.3		95.6, 47.2	
	Sn, Sp (%) test	99.0, 31.8		91.7, 45.4	
**Cut-off 90% Sn**		>0.433		>0.440	
	Sn, Sp (%) train	89.7, 75.0		89.7, 75.0	
	Sn, Sp (%) test	91.7, 77.3		83.3, 59.1	

Abbreviations: AIC= Akaike Information Criterion, BIC=Bayesian Information Criterion, Sn=Sensitivity, Sp=Specificity

[a] For each of the two fitted models, we calculated two cut-off points based on maximizing the specificity at two different levels of specificity (Sn=95%, Sn=90%). Optimal models were selected using function *dredge* form the R package *MuMIn*

SUPPLEMENTARY TABLE S5: Patient Demographics and Classification of Lung Cancer

		TRAINING SET		VALIDATION SET	
		**Lung Cancer (n=68)**	**Control (n=72)**	**Lung Cancer (n=24)**	**Control (n=22)**
**Gender**[a]					
	**Male**	55 (80.9%)	46 (63.9%)	20 (83.3%)	14 (63.6%)
	**Female**	13 (19.1%)	26 (36.1%)	4 (16.7%)	8 (36.4%)
**Age**[b]					
	**Median**	69.5	61	64	59.5
	**Range**	47-88	24-87	37-86	38-88
**Smoking status**[c]					
	**Yes**	63 (92.6%)	49 (68.1%)	20 (83.3%)	14 (63.6%)
	**No**	5 (7.4%)	23 (31.9%)	4 (16.7%)	8 (36.4%)
**Diagnosis**					
	**Healthy**		36 (50%)		8 (36.4%)
	**RI**		30 (41.7%)		11 (50%)
	**ILD**		6 (8.3%)		3 (13.6%)
	**NSCLC**	59 (86.8%)		21 (87.5%)	
	ADC	32 (47.1%)		12 (50%)	
	SCC	13 (19.1%)		7 (29.2%)	
	LCC	11 (16.2%)		2 (8.3%)	
	BAC	2 (2.9%)1			
	ND	(1.5%)			
	**SCLC**	9 (13.2%)		3 (12.5%)	
**Stage**					
	**NSCLC**				
	I	14 (23.7%)		5 (23.8%)	
	II	2 (3.4%)		1 (4.8%)	
	III	15 (25.4%)		6 (28.6%)	
	IV	28 (47.5)		9 (42.9%)	
	**SCLC**				
	Limited	3 (33.3%)		2 (66.6%)	
	Extended	6 (66.6%)		1 (33.3%)	

Abbreviations: NSCLC=Non Small Cell Lung Cancer, ADC=Adenocarcinoma, SqCC=Squamous Cell Carcinoma, LCC=Large Cell Carcinoma, BAC=Bronchioloalveolar Carcinoma, ND=Not Differentiated Carcinoma, SCLC=Small Cell Lung Cancer, RI=Respiratory Infection, ILD=Interstitial Lung Disease

[a] Gender distribution between cancer and controls statistically significant in the training set: *P*=0.037 (Fisher test)

[b] Statistically significant differences in age between cancer and controls in the training set: *P*=0.017 (Mann-Whitney U test)

[c] Smoking status distribution between cancer and controls statistically different: *P*<0.001 in training set (Fisher test)
